# Generation and use of functionalised hydrogels that can rapidly sample infected surfaces^☆^

**DOI:** 10.1016/j.mex.2022.101684

**Published:** 2022-04-17

**Authors:** Thomas Swift, Abigail Pinnock, Nagaveni Shivshetty, David Pownall, Sheila MacNeil, Ian Douglas, Prashant Garg, Stephen Rimmer

**Affiliations:** a University of Bradford, Bradford UK; b University of Sheffield, Sheffield UK; c L V Prasad Eye Institute, Hyderabad, India; d University of Bradford, Bradford UK; e University of Sheffield, Sheffield UK; f University of Sheffield, Sheffield UK; g L V Prasad Eye Institute, Hyderabad, India; h University of Bradford, Bradford UK

**Keywords:** Contact lens, Pathogen diagnosis, Medical device, Specificity, Amphotericin elisa, Polymyxin elisa, Highly branched polymers

## Abstract

This paper outlined our method for developing polymer-linked contact lens type materials for rapid detection and differentiation of Gram-positive, Gram-negative bacteria and fungi in infected corneas. It can be applied to both model synthetic or ex-vivo corneal models and has been successfully trialed in an initial efficacy tested animal study. First a hydrogel substrate for the swab material is selected, we have demonstrated selective swabs using a glycerol monomethacrylate hydrogel. Alternatively any commercial material with carboxylic acid functional groups is suitable but risks nonspecific adhesion. This is then functionalised via use of N-hydroxysuccinimide reaction with amine groups on the specified highly branched polymer ligand (either individually gram negative, gram positive or fungal binding polymers or a combination of all three can be employed for desired sensing application). The hydrogel is then cut into swabs suitable for sampling, used, and then the presence of gram positive, game negative and fungi are disclosed by the sequential addition of dyes (fluorescent vancomycin, fluorescein isothiocyanate and calcofluor white).

In summary this method presents:

Method to produce glycerol monomethacrylate hydrogels to minimize nonspecific binding

Methods of attaching pathogen binding highly branched polymers to produce selective hydrogel swabs

Method for disclosing bound pathogens to this swab using sequential dye addition

## Graphical abstract

**Figure:**
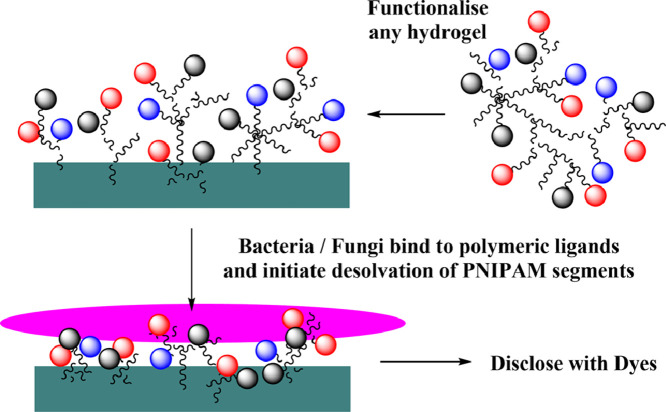

Specifications table

**Table:** 

**Subject Area**	Materials Science
**More specific subject area**	Polymer Chemistry and Microbiology
**Method name**	Functionalization of Hydrogels for Specified Detection of Microbes
**Name and reference of original method**	Highly branched PNIPAM polymers containing vancomycin or polymyxin functionalized end groups (but residual succinimide units for surface attachment) have previously been demonstrated to be attached to amine functional membranes and used to reduce infection burden in simulated wounds by Shepherd et al.[1] This work describes the efficacy and specificity of binding but offers no method of detecting or diagnosing infection, and it is specific to bacteria with no impact on fungal or mixed infection types.The vancomycin functionalization Elisa described within this report is a modification of the one we have previously outlined in the paper by Teratanatorn et al which outlined the use of the method for soluble polymer materials.[2]
**Resource availability**	This method will require access to both a chemical and a biological wet laboratory space equipped with glassware and reagents specified. Additionally, an ultrafilter, shaker, plate reader and microscope will be required for synthesis and characterization of the devices described herein alongside chemical analytical apparatus (NMR, FTIR, Size Exclusion Chromatography) for full chemical analysis of the polymer additives.

## Introduction

n warm, temperate climates there is an increased incidence of microbial keratitis, an infection of the cornea, making it one of the leading causes of vision loss in many countries. [1,2] Early and rapid diagnosis is imperative for effective and appropriate treatment. However, the remote locations of treatment centres means diagnosis is slow and often broad spectrum antibiotics are prescribed, contributing to an increase in antibiotic resistance. The aim of this study is to fabricate polymer-linked contact lens type hydrogels and use them for rapid detection of both Gram-positive, Gram-negative bacteria and fungi in infected cornea. The method is demonstrated using both rabbit and human ex-vivo corneas and has been replicated in animal safety trials and should be suitable for other skin or wound surface sampling as required.

This method of rapid detection and disclosure is of vital importance as there is approximately a 10-30 times greater incidence of corneal ulceration in developing countries than more industrialised, developed countries, [3] where work in agricultural settings and a high prevalence of home remedies can exacerbate infectious diseases. [4] The most commonly isolated bacterial species from corneal scrapings include *Staphylococcus aureus, Staphylococcus epidermidis, Streptococcus pneumonia* and *Pseudomonas aeruginosa* and the major fungal isolates include *Fusarium solani, Candida albicans* and *Aspergillus fumigatus.* [5], [6], [7] The key to effective management of disease is early diagnosis and appropriate treatment. However, early diagnosis in these regions of the world is difficult due to the remote locations of treatment centres. [8]

The gold standard for identifying the infecting organism is microbial culture. However, this can be slow because cultures must be performed at a central centre and the limited number of these centres mean that diagnosis is not rapid. [9] The result of this is that clinicians need to treat the infection immediately to preserve sight without any scientific indicators of the pathogen strain, and therefore the preferential treatment option. Therefore, broad spectrum antibiotics are given until the appropriate treatment is found which depends on the infecting organism. This high incidence of inappropriate treatment is associated with increased microbial resistance. [10] Consequently, there is a pressing need for a novel inexpensive and rapid detection system, suitable for use at remote treatment centres, that would allow early diagnosis and dictate the appropriate treatment course. [11] Several of these systems employ fluorescent dyes that specifically target gram positive [12] however it is a challenge to find fluorescent stains that can provide the level of discrimination for the vast array of pathogenic microbial species desired.

Our method achieves this by attaching a highly branched polymer additive functionalized with vancomycin (van), polymyxin (pmx) and amphotericin (amp) ligands to a hydrogel sheet that is cut into the size and shape suitable for placement on a human eye. The material must contain a high degree of amine functionality in order to form a strong bond with the binding polymer, but we have demonstrated how any carboxylic acid functional contact lens (such as any typical commercial hydrogel contact lens) can be used with a two-step modification although this may impact both the selectivity of the system and the choice of disclosing dyes. Clinical isolates of *S. aureus, P. aeruginosa* and *C. albicans* (1 × 10^8^) were directly incubated with polymer-linked lenses before being washed and imaged using a light or fluorescent microscope or finely minced and the numbers of viable bacteria enumerated. These strains were selected to be representative of the major broad categories of infectious pathogens in wound care.

We have outlined this method using both directly synthesized glycerol monomethacrylate (GMMA) Hydrogel modification (section 1) and commercial contact lenses (section 2). The first method builds on our published materials and provide the steps outlined to prepare highly specified detection of specific bacterial species whilst section 2 shows a relatively simple modification to the published methodology which would allow for functionalization of a vast array of other commercial hydrogel products to create pathogenic sensor materials – at the sacrifice of some specificity as described in the provided method validation data.

## Section 1: fabricated glycerol monomethacrylate hydrogel method

Glycerol monomethacrylate (GMMA) (5 g, 4.660 ml), Glycidyl methacrylate (GME)) (0.345 g, 0.321 ml) and ethylene glycol dimethacrylate (EGDMA) (0.206 g, 0.196 ml) were degassed via bubbling dry nitrogen through solution whilst stirring in isopropanol (2 ml) for twenty minutes. 2-hydroxy-2-methylpropiphenone (HMPP) (55 mg) was added and the solution degassed for a further five minutes before it was extracted using a glass syringe and directly injected into a quartz plate mould separated with a 0.5 mm PTFE gasket. The two quartz plates were laminated with poly (ethylene terephthalate) sheet, which was adhered to inner surfaces of the glass, to aid the release of the produced polymer sheet. To initiate polymerisation the mould was irradiated by a 400-w metal halide UV-A lamp for 3 minutes before being turned over and irradiated on the alternate side for a further 3 minutes. The cured hydrogel sheet was then removed and immersed in isopropanol. The hydrogel sheet was washed a total of five times with fresh isopropanol and left for at least 1 hour each time before being added to a 1,3-diaminopropane solution in isopropanol (20% v/v, 250 ml) solution for 48 hours, being inverted halfway through. It was then washed and immersed for 1 hour in isopropanol a further two times. The hydrogel was characterised by measurement of equilibrium water content (EWC = 61%, SD = 4%, n = 12). Fourier Transform Infrared spectroscopy (FTIR) was used to analyse for residual monomer leaching and the material was imaged using scanning electron microscopy.

Aminated hydrogels were exposed to HB-PNIPAM-X (50 mg), where X is either van, pmx or amp, dissolved in isopropanol (100 ml). The hydrogel sheets were immersed for 48 hours on a low-speed shaker with inversion after 24 hours When the polymers had reacted, the sheet was washed with isopropanol for one hour. The isopropanol was refreshed, left for a further hour. To deprotect the HB-PNIPAM-pmx (removal of FMoC groups) 20 ml of piperidine in isopropanol (20% v/v) was added to the hydrogel sheet for 48 hours before being washed in pure isopropanol for an hour, three further times. Polymer films were characterised by assessing equilibrium water content (EWC) (see Table 1) polymer loading by UV-absorbance and Vancomycin ELISA and FTIR. Full characterisation details are shown in the supporting information.

To produce a tri-functional hydrogel the aminated hydrogel discs (5 mm diameter) were exposed to a mixture of HB-PNIPAM-van (50 mg), HB-PNIPAM-pmx (100 mg) and HB-PNIPAM-amp (60 mg) dissolved in isopropanol (100 ml). Hydrogels containing other amounts are disclosed in the supporting information. These exposed discs are described as triple functional hydrogels in this work. The hydrogel sheet was left immersed in this mixture for 48 hours on a slow speed shaker and the hydrogel inverted halfway through. When the polymers had reacted, the sheet was washed with isopropanol for one hour. The isopropanol was refreshed, left for a further hour. To deprotect the HB-PNIPAM-pmx (removal of FMoC groups) 20 ml of piperidine in isopropanol (20% v/v) was added to the hydrogel sheet for 48 hours before being washed in pure isopropanol for an hour, three further times. Prior to use all hydrogels were washed three times in PBS and then incubated in media and hydrogels were characterised via the same methods shown above.

### Specific method protocol

**Raw materials:** Glycerol monomethacrylate (GMMA), Glycidyl methacrylate (GME), Ethylene glycol dimethacrylate (EGDMA), Isopropanol (IPA), 2-hydroxy-2-methylpropiphenone (HMPP), 1,3-diaminopropane and functionalized polymer.

**Protective clothing:** Wear a laboratory coat, Wear safety spectacles, wear protective rubber gloves

**Diagram 1:**
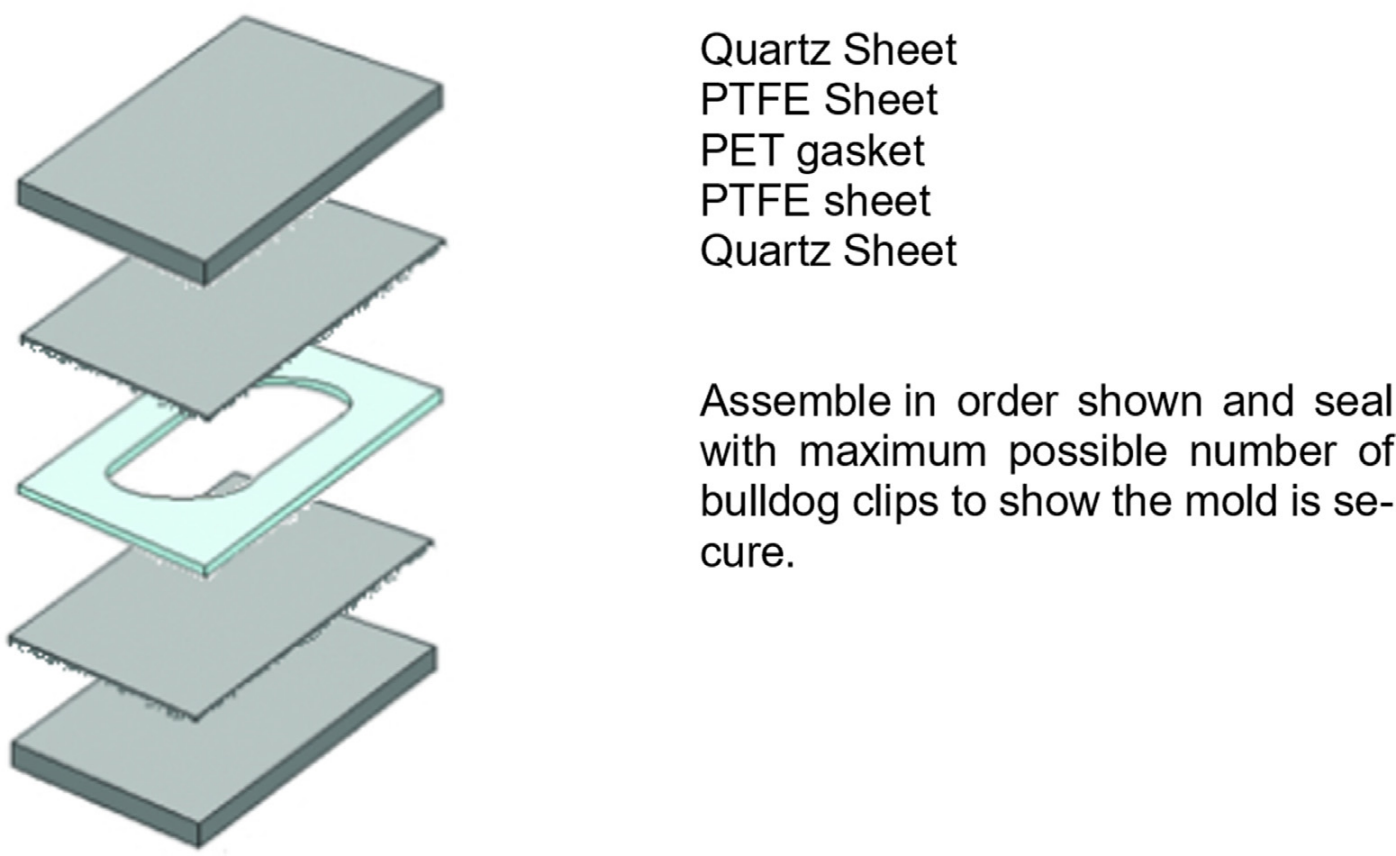
Assembly of Mold for Hydrogel synthesis.

**Equipment:** Quartz Plates x 2 (10 cm x 10 cm), PET gasket (0.5 mm) (10 cm x 10 cm, internal diameter 9 cm x 9 cm), PTFE sheets (cut to 10 cm x 10 cm), Dymax UV oven (400 W metal halide UV-A lamp), 5- 10 Bulldog clips, assemble mould following instructions in Diagram 1 and low speed shaker.

**Location:** Must be carried out in an extracted fume hood

**Procedure (Step 1):**

Into a round-bottomed flask, combine GMMA (5 g, 4.660 ml), GME (0.345 g, 0.321 ml) and EDGMA (0.206 g, 0.196 ml) with isopropanol (2 ml).
Add a magnetic stirrer bar and begin stirring.
Degas the solution by bubbling dry nitrogen through it for 20 minutes.
Add HMPP (55 mg).
Degas for a further 5 minutes.
Inject the monomer mixture into a mold consisting of two quartz plates lined with PET sheets, separated by a 0.5 mm PTFE gasket. Follow instructions on Diagram 1.
Irradiate the mold containing the monomer mixture using a 400W metal Halide UV-A lamp for 3 minutes on each side.
Remove the cured hydrogel sheet from the mold and place in isopropanol.
Wash the hydrogel sheet a total of 5 times in isopropanol for at least 1 hour per washing.
Add the hydrogel to a solution of 1,3-diaminopropane in isopropanol (20% v/v, 250 ml) for 24 hours.
Flip over the hydrogel sheet and leave in the diamine solution for a further 24 hours.
Remove the hydrogel from the solution and wash twice in pure isopropanol twice for a minimum of 1 hour.
Check for the presence of residual monomer by soaking a sample of the hydrogel in methanol for 12 hours then carrying out GC analysis on the methanol supernatant.
Determine the equilibrium water content (steps outlined separately in supporting information) of the hydrogel by sampling it.

**Step 1 Pass/ fail criteria:** Films are stable and do not decompose and EWC lies between 55 and 70.

**Procedure (Step 2):**

In a sealable plastic container that is large enough to fit a hydrogel sheet flat against the bottom, Dissolve 50 mg vancomycin functional polymer, 100 mg polymyxin functional polymer and 60 mg amphotericin functional polymer in 100 ml IPA.
Place a hydrogel sheet (SOP 24) into the polymer solution.
Place the plastic container on top of a shaker and shake for 24 hours at the lowest speed. Remove the gel from the solution and flip over. Replace on the shaker.
Shake at minimum speed for 24 hours.
Remove the gel from the polymer solution and place in a container containing pure IPA for 1 hour.
Repeat the washing in step 6.
Deprotect the hydrogel (steps outlined separately below).
Measure the equilibrium water content and FTIR spectrum

**Step 2 Pass Criteria**: Films are stable and do not decompose, EWC lies between 40 and 65.

This procedure above outlines the creation of a triple functionalized polymer where the loading of vancomycin, polymyxin and amphotericin functional material has been optimized to give significant and detectible binding of all three strains of pathogen. The loadings of polymer can be easily altered or you can specify just one or two of the three strains of drug functionalized branched polymer to provide increased specificity to the diagnostic.

**Hydrogel Deprotection Protocol Expanded:**

Add 20 ml of piperidine in isopropyl alcohol (20% v/v) to a sealable container.
Add the functionalized hydrogel sheet and ensure that it is fully submerged.
Leave overnight
Flip the sheet and leave overnight.
Remove the hydrogel from the solution and place in a container containing pure IPA for a minimum of 1 hour to wash the gel.
Repeat the washing process two more times

This is a necessary process to remove any remaining Fmoc protecting groups on the polymyxin functionalized polymer. It can be easily adapted for the suspended polymer solution if you wish to deprotect the polymer and use separately from the hydrogel device – however once deprotected the polymer cannot be later attached to a surface the succinimide reaction will destroy the polymyxin drug functionality. The polymyxin polymer itself is perfectly soluble in methanol, chloroform or water at low temperature after the Fmoc protecting groups have been removed and so can be separated from the raw polymer material by simple filtration.

### Packaging of discs

We have had success storing and shipping these materials internationally by following the following procedure to ensure both product stability and stability are maintained:

Soak the hydrogel sheets in ethanol for a minimum of 1 hour.
Select the appropriate size of cork borer (3 mm, 5 mm or 10 mm depending on target profile. For animal trial experiment use smaller size dependent on instructions; for human clinical trials use 10 mm size).
Wipe a cutting board with 70% ethanol solution to disinfect.
Use the cork borer to cut the desired number of hydrogel disks from the sheet.
Place each hydrogel disk in a separate bijou container containing IPA.
Label each bijou container with the sample code, date and number (e.g., 1 of 6, 2 of 6 etc.)

On arrival at destination the following steps were undertaken to unpack and prepare them for longer term storage.

For polymer (powder) samples:

Place polymers received freeze-dried directly into -20°C storage until required
For use in assays, allow the polymers in glass vials to equilibrate to room temperature before opening (approximately half an hour)
Do not leave out of the freezer for longer than one hour

For hydrogel Samples on arrival:

Wash hydrogels, received in polymer solution, in isopropyl alcohol (IPA) twice and store in IPA at 4°C
Before use in biological assays, wash the hydrogels (approx. 3 × 2cm (for 1 experiment) 2 times in IPA then 3 times in PBS (5-minute washes each)
If additional hydrogel is removed and washed just before an assay and is subsequently not used, return it to a labelled glass vial containing IPA and store 4°C so it is clear which hydrogel has previously been washed in case this affects assays. Make a note of what ‘pot’ the hydrogel is taken from before each assay. Treat previously washed hydrogel as step 2 when using in subsequent experiments.
Do not leave out of the fridge for longer than half an hour

**Binding Studies (Testing the Functionality of Each Batch)**

**Culture of bacteria and fungi**

For rabbit corneas, laboratory strains of *S. aureus* (S-235), *P. aeruginosa* (SOM-1), *C. albicans* (SC5314) and *F. solani* strain (NCPF 2699), purchased from the National Collection of Pathogenic Fungi (UK), were used. For human corneas, ATCC cultures of *S. aureus* (25923), *P. aeruginosa* (27853), *C. albicans* (90028) and, *F. solani* CBS-132315 (Central Bureau Voor chimmcultures (CBS), Netherlands) were used. All bacterial and fungal strains were cultured on brain-heart infusion (BHI) agar at 37°C overnight and then maintained at 4°C. For use in experiments one colony was sub-cultured from agar into BHI broth and incubated overnight at 37°C. Stationary-phase microbes were used in rabbit cornea experiments. For human corneal experiments, on the day of corneal inoculation, a fresh broth was inoculated, and exponential-phase bacteria/fungi were used based on predetermined growth curves.

### Biological testing of materials

A sample of materials from each batch produced during this study was kept for biological testing – both to ensure sterility (as a means of fully sterilizing the product post modification could not be found which would not negatively impact the functionality of the polymer coating) and functionality. Some of the standard methods employed are listed below.

To evaluate binding of microorganisms to functionalised polymer hydrogels the following experiments were conducted:

a) in-vitro interaction of microorganisms to individual polymer-linked hydrogels.
b) Interaction and binding of organisms to hydrogel with all three functionalised polymers.
c) Assessment of the limit of attachment of microbes.
d) Determination of time duration for which the hydrogel needs to be placed on the cornea for optimal attachment.
e) Assessment of safety and efficacy of the triple hydrogel in-vivo in rabbits

10^8^ fluorescein isothiocyanate (FITC) labelled *S. aureus, P. aeruginosa* or *C. albicans* were incubated in-vitro with vancomycin-, polymyxin- or amphotericin B-functionalised polymers tagged on GMMA hydrogels respectively or triple hydrogels (all three agents) discs of 5 mm diameter for 1 hour. Hydrogels were washed 3 times with PBS, then imaged using a fluorescence microscope (Axiovert 200M, Zeiss). 8 fields of view were imaged and the number of organisms attaching to the hydrogels per field of view were analyzed using Image J and the imaging software AxioVision Rel. 4.8 in UK and ProgRes CapturePro 2.5 software (Jenoptik) in India. The number of organisms bound/attached to the functionalized hydrogels were compared with a non-functionalized hydrogel.

Single and triple functionalised hydrogels were placed for 60 minutes onto rabbit and human corneas that had been infected with 10^8^ *S. aureus, P. aeruginosa* or *C. albicans*. Hydrogels were picked up with sterile forceps, washed twice with PBS and stained with fluorescent dyes. Prior to staining with fluorescent Vancomycin or FITC, hydrogels were reacted with 0.1% periodic acid (Sigma) for 10 min, washed twice with PBS and then incubated with Schiff's reagent for 10 min before washing twice again. Hydrogels were incubated for 10 minutes with vancomycin Bodipy®FL conjugate (2 µg ml^−1^; FL-Vanc; ThermoFisher) for visualisation of Gram-positive (*S. aureus*) organisms, with FITC (0.5 mg ml^−1^) for Gram-negative organisms (*P. aeruginosa*) and with Calcofluor white using a 1:1 solution of Calcofluor white ready to use solution and 10% potassium hydroxide for visualisation of fungi. After incubation, the hydrogels were washed 3x in PBS and viewed under fluorescent microscope.

To assess the sensitivity of the functionalised hydrogels increasing numbers of *S. aureus, P. aeruginosa* or *C. albicans* were incubated in-vitro with triple-functionalised hydrogels for 1 hour. The hydrogels were washed, and the total ATP content determined using the ENLITEN® ATP assay kit according to the manufacturer's instructions. In another set of experiments increasing numbers of each organism were incubated in-vitro with triple functionalised hydrogels for 1 hour. Hydrogels were washed and then examined with a fluorescence microscope and the number of organisms per field of view counted. The data were compiled as mean + SD of 8 fields of view per hydrogel from at least 3 independent experiments.

Optimal time measurements for hydrogel placement were carried out using our ex-vivo cornea infection model described earlier. Human corneas were mono-infected with *S. aureus, P. aeruginosa* or *C. albicans*. Triple functionalized hydrogels were placed on to these infected corneas and left in place and it was found that a period of 30 minutes was sufficient length to bind sufficient numbers of micro-organisms from the ex-vivo lens that provided a statistically significant outcome under analysis.

A three step process was found to identify the three targeted strains of bacteria for each contact lens using fluorescent vancomycin,

**Detection of *S. aureus* using fluorescent vancomycin**

Grow an overnight BHI broth culture of *S. aureus* at 37°C
Count the number of cells and adjust the number to 10^8^ in 1 ml PBS
Add 100 µl of bacteria to wounded (using scalpel blade no. 22 to make 3 horizontal and 3 vertical slashes across the surface) ex vivo corneas (using a metal ring to ensure a tight seal)
Incubate overnight at 37°C
Wash corneas once with PBS
Cut a hydrogel disc of 1 cm diameter using a cork borer and place onto the surface of the cornea for 1 hour
Remove hydrogel and place into a 24 well plate containing 1 ml 1 % (w/v) periodic acid for 10 min
Wash with copious tap water (rinse in petri dish containing 50 ml water or under a running tap) for 5 min
Place into a 24 well plate containing 1 ml Schiff's reagent for 10 min
Wash with copious tap water (rinse in petri dish containing 50 ml water or under a running tap) for 5 min
Place into a 24 well plate containing 1 ml of 2 µgml^−1^ fluorescent vancomycin
Wash with copious tap water (rinse in petri dish containing 50 ml water or under a running tap) for 5 min
Visualise staining using a fluorescent microscope

**Total time for the procedure is approx. 55 minutes**

**Detection of *P. aeruginosa* using FITC**

Grow an overnight BHI broth culture of *P. aeruginosa* at 37°C
Count the number of cells and adjust the number to 10^8^ in 1 ml PBS
Add 100 µl of bacteria to wounded (using scalpel blade no. 22 to make 3 horizontal and 3 vertical slashes across the surface) ex vivo corneas (using a metal ring to ensure a tight seal)
Incubate overnight at 37°C
Wash corneas once with PBS
Cut a hydrogel disc of 1 cm diameter using a cork borer and place onto the surface of the cornea for 1 hour
Remove hydrogel and place into a 24 well plate containing 1 ml 1 % (w/v) periodic acid for 10 min
Wash with copious tap water (rinse in petri dish containing 50 ml water or under a running tap) for 5 min
Place into a 24 well plate containing 1 ml Schiff's reagent for 10 min
Wash with copious tap water (rinse in petri dish containing 50 ml water or under a running tap) for 5 min
Place into a 24 well plate containing 1 ml of 1mg ml^−1^ FITC in 0.05M sodium carbonate and 0.1M sodium chloride solution at 4°C for 1 hour
Wash with copious tap water (rinse in petri dish containing 50 ml water or under a running tap) for 5 min
Visualise staining using a fluorescent microscope

**Detection of *C.albicans* using calcofluor white**

Grow an overnight BHI broth culture of *C. albicans* at 37°C
Count the number of cells and adjust the number to 10^8^ in 1 ml PBS
Add 100 µl of fungi to wounded (using scalpel blade no. 22 to make 3 horizontal and 3 vertical slashes across the surface) ex vivo corneas (using a metal ring to ensure a tight seal)
Incubate overnight at 37°C
Wash corneas once with PBS
Cut a hydrogel disc of 1 cm diameter using a cork borer and place onto the surface of the cornea for 1 hour
Remove hydrogel and place into a 24 well plate containing 500 µl 10 % (w/v) potassium hydroxide and 500 µl calcofluor white for 2 min
Wash with copious tap water (rinse in petri dish containing 50 ml water or under a running tap) for 5 min
Visualise staining using a fluorescent microscope

**Total time for procedure is approx. 20 minutes**

**Fig. 1:**
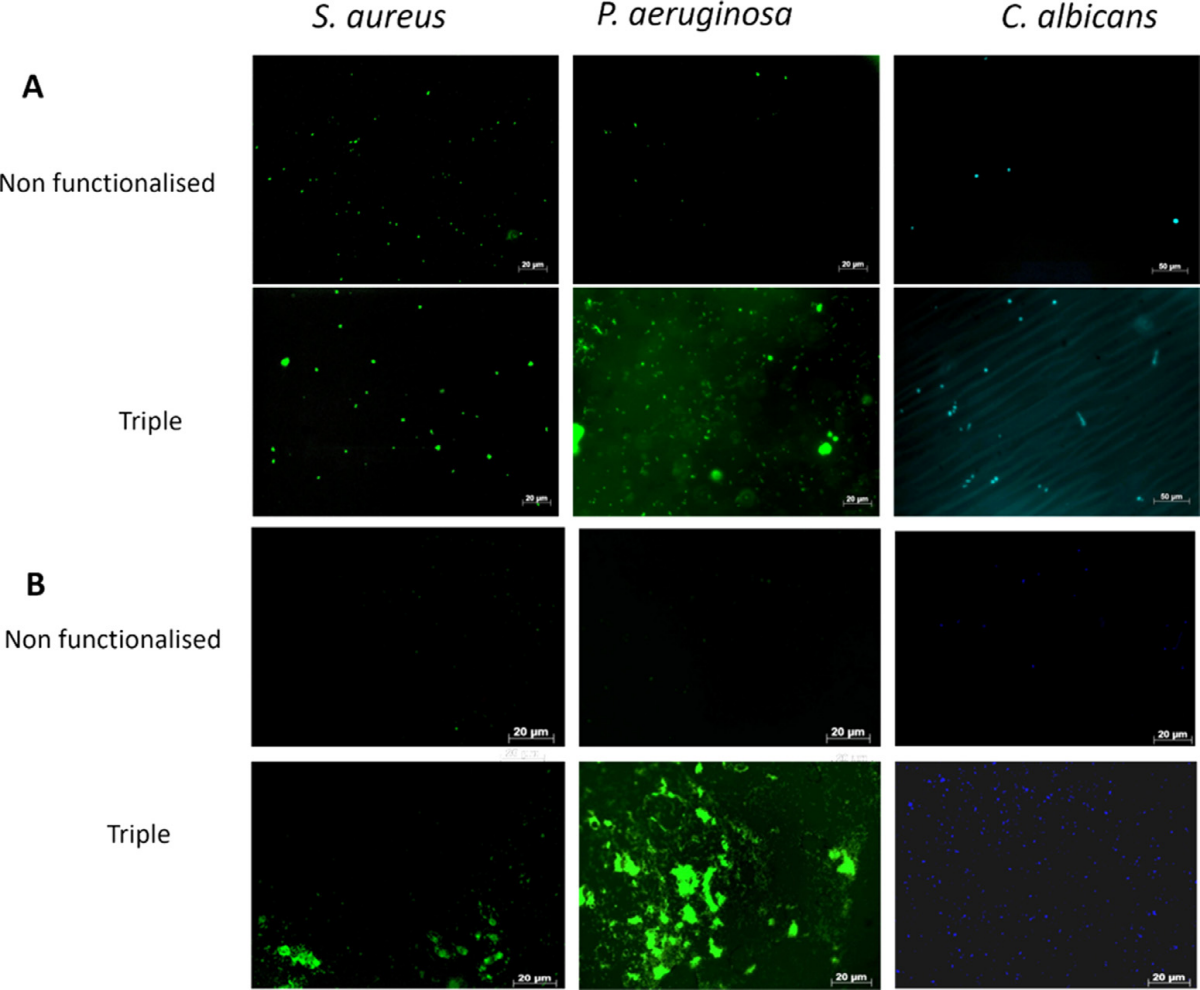
*S. aureus, P. aeruginosa* and *C. albicans* infections detected using a non functionaliszed and triple functionalized bacterial hydrogel. *Ex vivo* rabbit (A) /Human (B) corneas were infected with *S. aureus, P.aeruginosa* or *C.albicans* for 24 hours, washed and exposed to a dual functionalised bacterial hydrogel for 1 hr. Hydrogels with *S. aureus* were detected using fluorescent vancomycin and hydrogels with *P.aeruginosa* were detected using FITC. Both hydrogels were blocked using periodic acid and Schiff's reagent prior to staining. Hydrogels with *C.albicans* were detected using Calcofluor White. Images show *S. aureus, P.aeruginosa* and *C.albicans* bound to hydrogels removed from infected *ex vivo* corneas.

Using these dyes it is possible to sequentially test for all three species as shown in our example results in Fig. 1.

Fig. 2

**Fig. 2:**
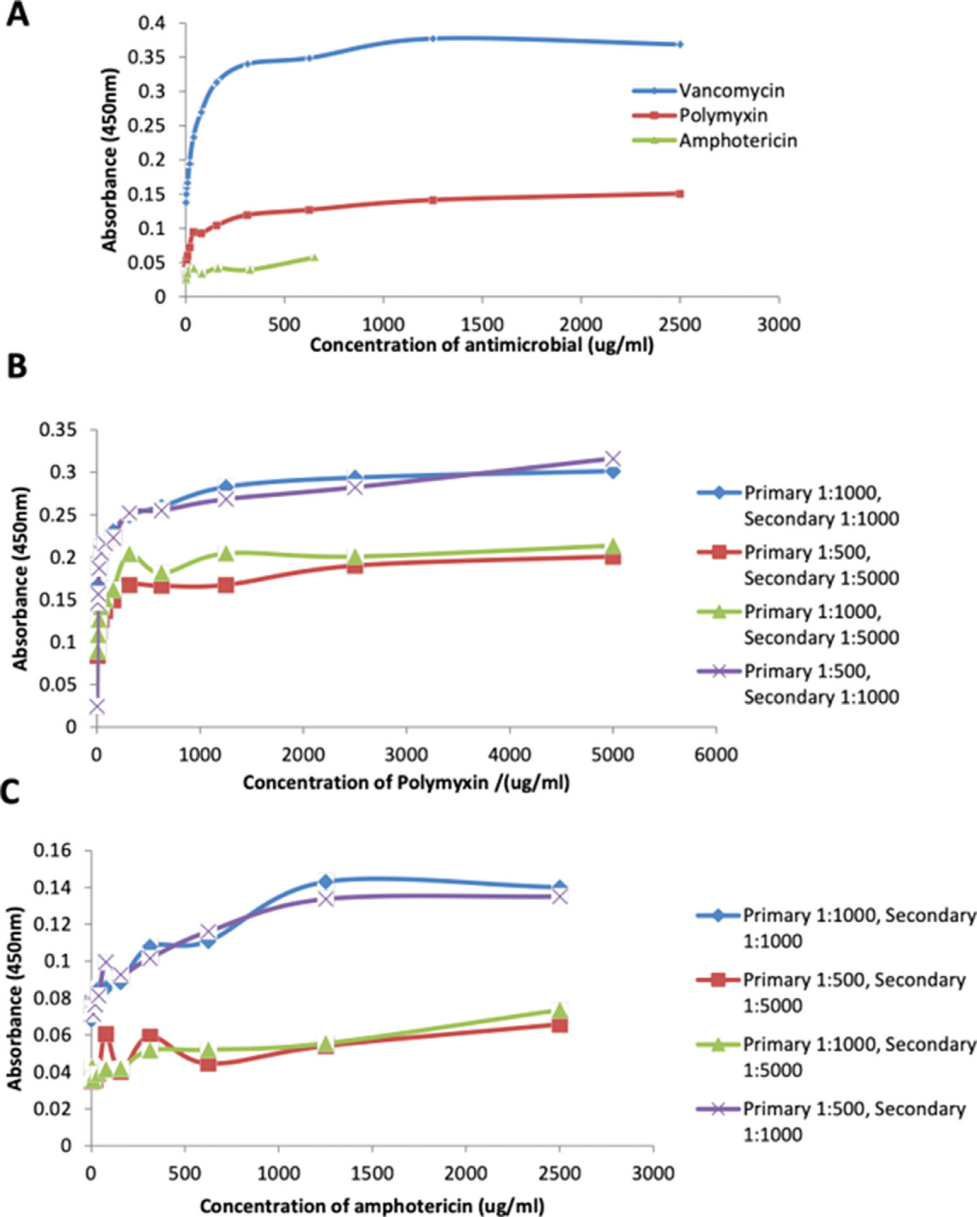
ELISA standard curves for vancomycin (A), polymyxin (B) and amphotericin (C). Vancomycin, polymyxin and amphotericin standard curve ELISAs were performed as described in Fig. 5. Initial experiments (A) used primary antibodies at 1:1000 and secondary antibodies at a concentration of 1:5000. Where the vancomycin ELISA showed a good standard curve, the curves for polymyxin and amphotericin required further optimisation of antibody concentrations. For polymyxin (B) the standard curve ELISA suggests that using a concentration of primary antibody of 1:1000 and secondary antibody of 1:1000 the standard curves will be comparable to the vancomycin standard curve that has previously been optimised. For amphotericin (C) further work is required to increase the plateau of the standard curve to approx. 0.3 OD. This was done by increasing the concentration of the secondary antibody.

Scheme 1

As an alternative to bacterial testing ELISA studies product control studies were carried out to determine the functionalization's for hydrogels. There were 3 different protocols to determine the functionalization of vancomycin, polymyxin and amphotericin respectively:

**Vancomycin ELISA for Hydrogels**
To a high binding ELISA plate add 100 µl monoclonal mouse anti-vancomycin (1:500 dilution in 35 mM NaHCO_3_, 15 mM Na_2_CO_3_ and 3mM NaN_3_ in distilled water) and cover the plate with adhesive plastic
Incubate the plate at 4°C overnight
Wash the plate 4 times with 200 µl PBS-Tween 20 (1:200,000 dilution of Tween: PBS) (PBS-T)
Add 200 µl of blocking buffer (5 % bovine serum albumin (BSA) in PBS-T) in order to block the non-specific binding sites on the coated well
Cover the plate with adhesive plastic and incubate at 4°C overnight
Wash the plate with 200 µl of PBS-T 4 times
Prepare standard vancomycin solutions (1:2 dilutions (in mg ml^−1^), e.g., 2.5, 1.25, 0.625, 0.313, 0.156, 0.078, 0.039, 0.019, 0.0097, 0.0048, 0.0024, 0 in 1 % BSA-PBS-T)
Cover the plate with adhesive plastic and incubate at 4°C overnight
In a U bottomed Eppendorf tube fill to the top (approx. 2 ml) with 5 % BSA-PBS-T and place 0.5 cm diameter hydrogel disc (one disc per tube) and incubate overnight at 4°C
Wash the plate and hydrogels with washing buffer 4 times
Add 100 µl to the wells (standards) or 300 µl to the 2.0ml tube (hydrogel pieces) of the monoclonal mouse anti-vancomycin detector antibody (1:5,000) with 1% BSA-PBS-T
Cover the plate with adhesive plastic and incubate at room temperature for 2 hours
Wash the plate with washing buffer 4 times
Add 100 µl to the wells (standards) or 300 µl to the 2.0ml tube (hydrogels) of HRP-conjugated antibody diluted to 1:3,000 with 1% BSA-PBS-T
Cover the plate with adhesive plastic and incubate at room temperature for 2 hours
Wash the plate with washing buffer 4 times
Dissolve TMB substrate tablet in 25 ml of 0.05 M phosphate-citrate buffer (pH 5.0)
Add 10 µl of 30 % H_2_O_2_ into TMB solution
Add 100 µl of TMB solution to each well of the plate or 200 µl to the 1.5ml tube ensuring all hydrogel piece is covered with solution
After sufficient colour development add 100 µl or 200 µl stop solution (1 M H_2_SO_4_) (1:1 TMB: stop solution)
Measure the absorbance of each well at 450 nm
Calculate the concentration of samples from the standard curve
**Polymyxin ELISA for Hydrogels**
To a high binding ELISA plate add 100 µl monoclonal mouse anti-polymyxin (1:1000 dilution in 35 mM NaHCO_3_, 15 mM Na_2_CO_3_ and 3mM NaN_3_ in distilled water) and cover the plate with adhesive plastic
Incubate the plate at 4°C overnight
Wash the plate 4 times with 200 µl PBS-Tween 20 (1:200,000 dilution of Tween: PBS) (PBS-T)
Add 200 µl of blocking buffer (5 % bovine serum albumin (BSA) in PBS-T) in order to block the non-specific binding sites on the coated well
Cover the plate with adhesive plastic and incubate at 4°C overnight
Wash the plate with 200 µl of PBS-T 4 times
Prepare standard polymyxin solutions (1:2 dilutions (in mg ml^−1^), e.g., 2.5, 1.25, 0.625, 0.313, 0.156, 0.078, 0.039, 0.019, 0.0097, 0.0048, 0.0024, 0 in 1 % BSA-PBS-T) and add to the plate
Cover the plate with adhesive plastic and incubate at 4°C overnight
In a U bottomed Eppendorf tube fill to the top (approx. 2 ml) with 5 % BSA-PBS-T and place 0.5 cm diameter hydrogel disc (one disc per tube) and incubate overnight at 4°C
Wash the plate and hydrogels with washing buffer 4 times
Add 100 µl to the wells (standards) or 300 µl to the 2.0ml tube (hydrogel pieces) of the biotinylated monoclonal mouse anti-polymyxin antibody (1:1,000) with 1% BSA-PBS-T
Cover the plate with adhesive plastic and incubate at room temperature for 2 hours
Wash the plate with washing buffer 4 times
Add 100 µl to the wells (standards) or 300 µl to the 2.0ml tube (hydrogels) of ABC reagent prepared according to manufacturers instructions (http://vectorlabs.com/uk/vectastain-elite-abc-kit-universal.html?SID=743e1e74ad49a56ec2eb2bc61adcbb86)
Cover the plate with adhesive plastic and incubate at room temperature for 30 min
Wash the plate with washing buffer 4 times
Dissolve TMB substrate tablet in 25 ml of 0.05 M phosphate-citrate buffer (pH 5.0)
Add 10 µl of 30 % H_2_O_2_ into TMB solution
Add 100 µl of TMB solution to each well of the plate or 200 µl to the 2.0ml tube ensuring all hydrogel piece is covered with solution
After sufficient colour development add 100 µl or 200 µl stop solution (1 M H_2_SO_4_) (1:1 TMB: stop solution)
Measure the absorbance of each well at 450 nm
Calculate the concentration of samples from the standard curve
**Amphotericin ELISA for Hydrogels**
To a high binding ELISA plate add 100 µl polyclonal anti-amphotericin (1:1000 dilution in 35 mM NaHCO_3_, 15 mM Na_2_CO_3_ and 3mM NaN_3_ in distilled water) and cover the plate with adhesive plastic
Incubate the plate at 4°C overnight
Wash the plate 4 times with 200 µl PBS-Tween 20 (1:200,000 dilution of Tween: PBS) (PBS-T)
Add 200 µl of blocking buffer (5 % bovine serum albumin (BSA) in PBS-T) in order to block the non-specific binding sites on the coated well
Cover the plate with adhesive plastic and incubate at 4°C overnight
Wash the plate with 200 µl of PBS-T 4 times
Prepare standard amphotericin solutions (1:2 dilutions (in mg ml^−1^), e.g., 2.5, 1.25, 0.625, 0.313, 0.156, 0.078, 0.039, 0.019, 0.0097, 0.0048, 0.0024, 0 in 1 % BSA-PBS-T) and add to the plate
Cover the plate with adhesive plastic and incubate at 4°C overnight
In a U bottomed Eppendorf tube fill to the top (approx. 2 ml) with 5 % BSA-PBS-T and place 0.5 cm diameter hydrogel disc (one disc per tube) and incubate overnight at 4°C
Wash the plate and hydrogels with washing buffer 4 times
Add 100 µl to the wells (standards) or 300 µl to the 2.0ml tube (hydrogel pieces) of the biotinylated polyclonal anti-amphotericin antibody (1:500) with 1% BSA-PBS-T
Cover the plate with adhesive plastic and incubate at room temperature for 2 hours
Wash the plate with washing buffer 4 times
Add 100 µl to the wells (standards) or 300 µl to the 2.0ml tube (hydrogels) of ABC reagent prepared according to manufacturers instructions (http://vectorlabs.com/uk/vectastain-elite-abc-kit-universal.html?SID=743e1e74ad49a56ec2eb2bc61adcbb86)
Cover the plate with adhesive plastic and incubate at room temperature for 30 min
Wash the plate with washing buffer 4 times
Dissolve one TMB substrate tablet in 25 ml of 0.05 M phosphate-citrate buffer (pH 5.0)
Add 10 µl of 30 % H_2_O_2_ into TMB solution
Add 100 µl of TMB solution to each well of the plate or 200 µl to the 2.0ml tube ensuring all hydrogel piece is covered with solution
After sufficient colour development add 100 µl or 200 µl stop solution (1 M H_2_SO_4_) (1:1 TMB:stop solution)
Measure the absorbance of each well at 450 nm
Calculate the concentration of samples from the standard curve

## Example results

These ELISA's can also be modified to test the concentration of the active drug on the powder polymer additive with minor modifications.

### Section 2: commercial contact lens modification

Commercially available single use contact lenses underwent a three-step modification to attach vancomycin (van) and polymyxin (pmx) functionalised highly branched poly(N-isopropylacrylamide). The carboxylic acid groups within the contact lenses were first modified with excess ethandiamine then the free amine groups from monosubstituted ethandiamine were reacted, forming amide linkages, with remaining carboxylic acid groups on the highly branched polymers.

**Fig. 3:**
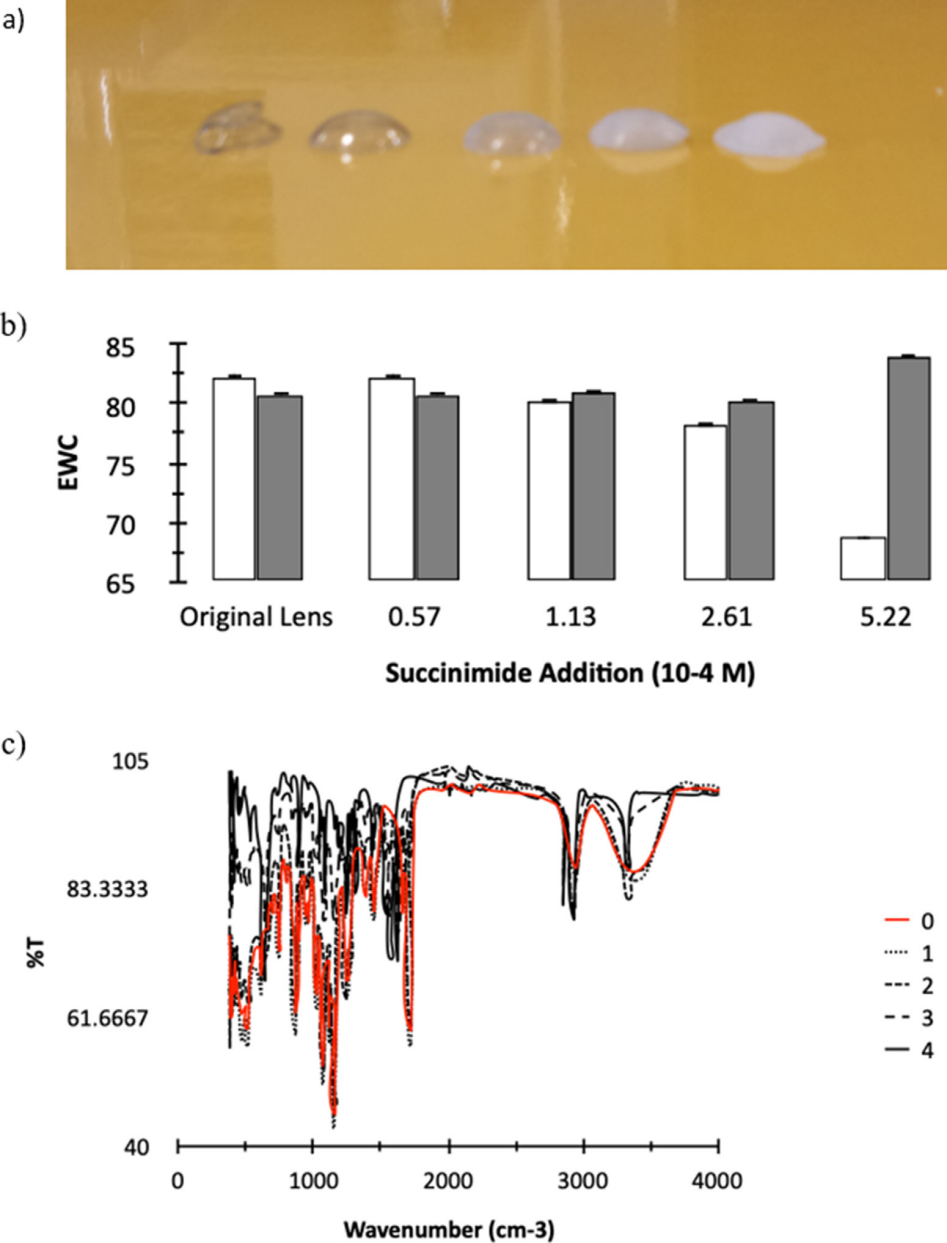
Modification of contact lenses with increasing succinimide modification. a) Visual comparison of succinimde lenses, EWC of succinimide (clear) and diamine (grey) modified lenses b) EWC of contact lens following succinimide addition (clear) and additional diamine grafting (grey). C) FTIR data of the modification contact lenses at 4 levels of succinimide addition.

Modification of contact lenses was carried out using autoclaved water and in a sterilised environment. Contact Lenses (Biomedics 1 day Extra, Ocufilcon D, Coopervision) were prepared for polymer attachment via a two stage modification via an excess of N-(3-Dimethylaminopropyl)-N′-ethylcarbodiimide hydrochloride (EDC) (or N',N'-dicyclohexyl carbodiimide (DCC) in dimethyl formamide (DMF)) and N-Hydroxysuccinimide (5 × 10^−4^ M) and then mixed with ethylenediamine (0.017 M) and left to react for 24 hours. These contact lenses were washed to remove the supernatant and then soaked in a dilute solution of partially modified antimicrobial polymer for 24 hours. Contact lens functionalised with HB-PNIPAM-pmx were treated with 20% piperidine (5 ml) to remove FMoC blocking groups and washed prior to bacterial detection. The modification of the contact lens can be observed via both the changing opacity of the contact lens (with increasing succinimide modification it became entirely opaque – Fig. 3A), but also a change in the volume of water the hydrogel can absorb (Fig. 3B) and the decrease of FTIR peaks at 1720 and 3300 cm^−1^ (Fig. 3C - signifying reduction of the methacrylic acid loading at the surface of the contact lens)

Contact lens modification was carried out in aqueous solution using EDC and N-hyroxysuccinimide in a 1: 0.5 molar ratio (0.0013 moles). 1 ml of solution was added to contact lens and left for 24 hours, rinsed and then reacted with 0.017 M ethylene diamine in 3 ml aqueous solution at pH 10. After 24 hours these were rinsed again before mixing with polymer in a dilute aqueous solution. Two separate batches of vancomycin modified polymer were used (7.3 and 7.5 mg), and others were combined with succinimide-modified polymer with no antimicrobial functionality (5.8 mg). The last batch was mixed with a fluorescein containing polymer to allow for leaching tests of the contact lens to be carried out (5.0 mg).

Clinical isolates of *S. aureus* and *P. aeruginosa* (1 × 10^8^) were FITC labelled and directly incubated with polymer-linked lenses for 1 hour at 37°C. Lenses were washed and imaged using a light or fluorescent microscope or finely minced and the numbers of viable bacteria recovered enumerated. Cornea or corneal epithelial cell viability was assessed using Alamar Blue after 48h exposure to varying concentrations of soluble van and pmx polymer (1 - 5 mg ml^−1^). After application and removal of polymer-functionalised lenses, their ability to bind and remove *S.aureus* or *P.aeruginosa* from the infected ex vivo corneas was assessed by light microscopy and histology.

**Fig. 4:**
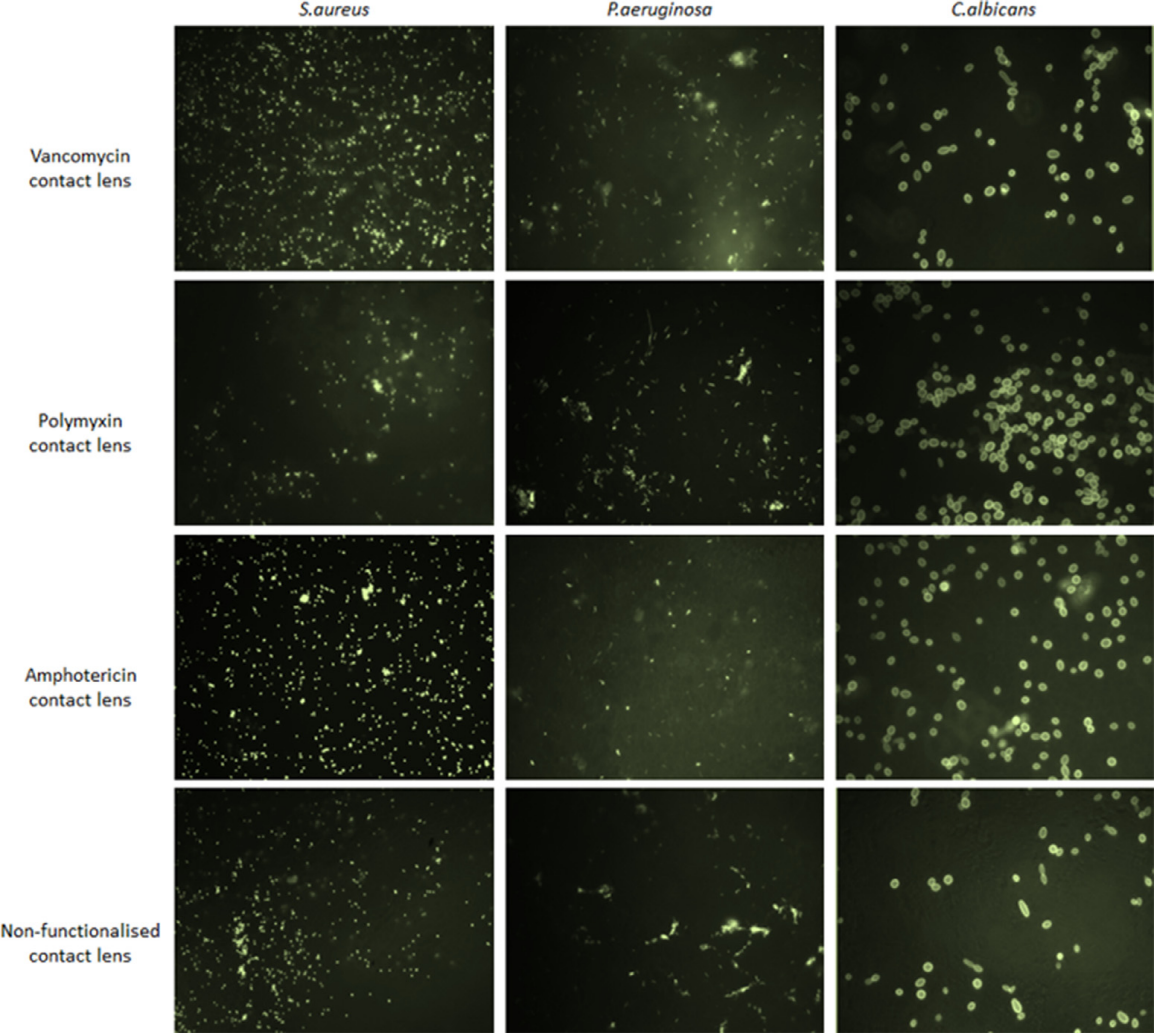
Commercial contact lenses show non-specificity of binding for *S.aureus, P.aeruginosa* and *C.albicans*. 10^8^ FITC-labelled *S.aureus, P.aeruginosa* and *C.albicans* were incubated with vancomycin polymer-functionalised, polymyxin polymer-functionalised, amphotericin polymer-functionalised and non-functionalised (diamine) contact lenses for 1 hr, washed and visualised under a fluorescence microscope.

Via this protocol we have been able to demonstrate that almost any suitable hydrogel material may be functionalized with the highly branched polymer additive to increased affinity to bacterial or fungal isolates. However, in our studies we have found whilst the resultant modified contact lens attached a high amount of the desired bacteria the binding was far less non-specific than that provided by the GMMA hydrogel. Depending on the target use of the product (desired specificity) it is a viable alternative to fabrication of custom made hydrogels as outlined in the steps above. If the substrate (hydrogel) material used is sufficiently absorbant to bacteria then non-specific binding may be observed as shown In Fig. 4.

**Fig. 5:**
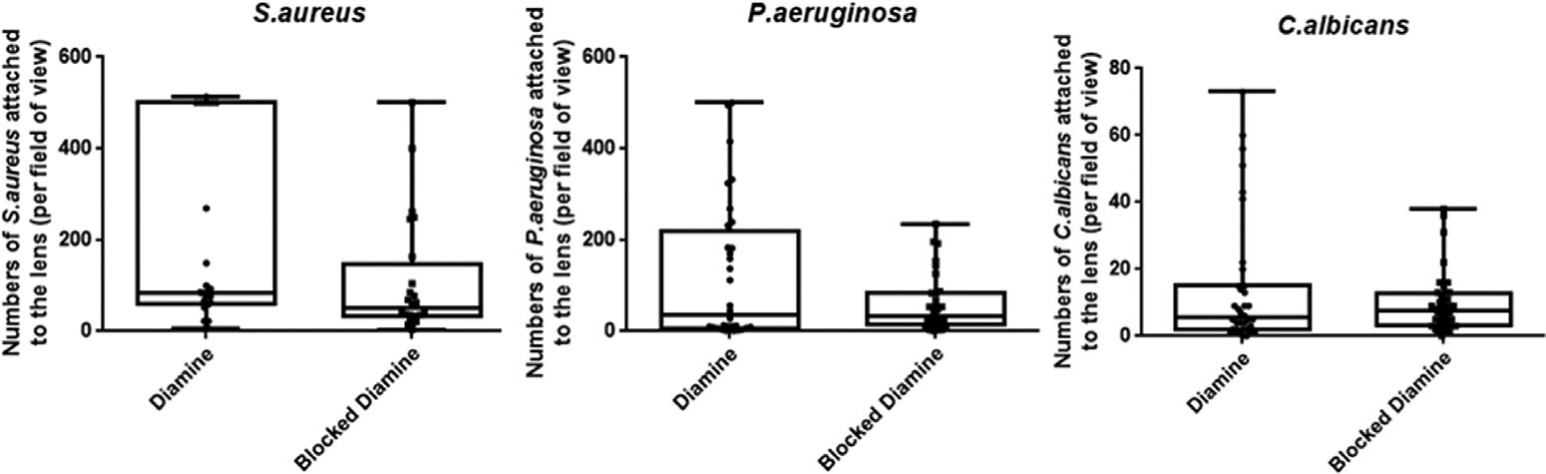
Blocking commercial contact lenses with acetic acid and EDC showed some reduction in non-specificity of bacterial and fungal binding, but not considerably. 10^8^ FITC-labelled *S. aureus, P.aeruginosa* and *C.albicans* were incubated with commercial contact lenses treated with diamine (not functionalised with polymer) or contact lenses treated with diamine and then blocked with acetic acid and EDC (no polymer), for 1 hr, washed and visualised under a fluorescence microscope.

When the non-specific binding of this system was discovered we suspected it was due to the residual diamine groups and so work was undertaken to block the diamine by addition of 100% acetic acid (1 ml) and EDC (5 mg) to reduce nonspecific bacteria adherence however this was not entirely successful (Fig. 5).

**Fig. 6:**
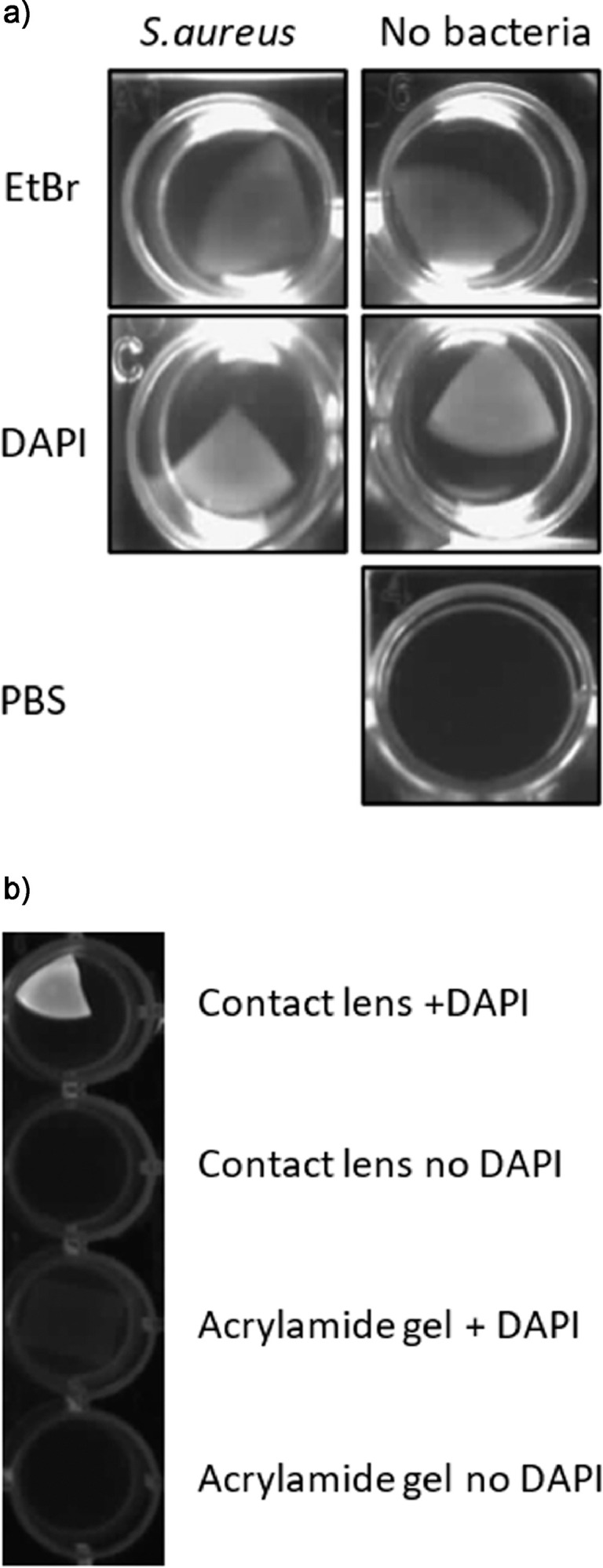

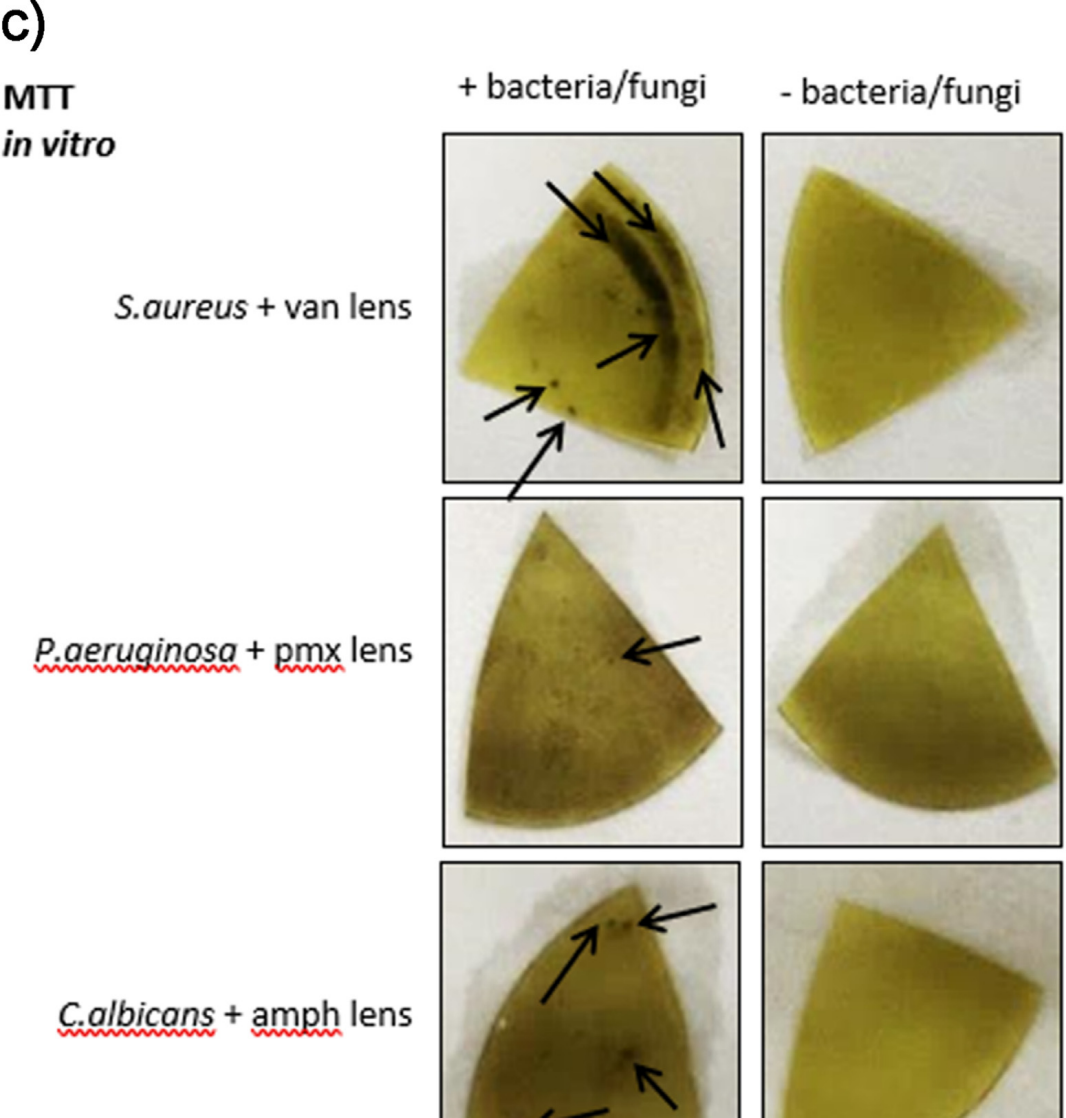
Studies to determine method of detecting pathogens bound to contact lens surfaces. a) High background staining of contact lenses by ethidium bromide and DAPI. 10^8^*S.aureus* were incubated with vancomycin polymer functionalised lenses for 1 hour. After one hour the lenses were washed 3x in PBS and incubated with EtBr, DAPI or PBS for 30 minutes and imaged using a UV light box. Contact lenses, both with and without bacteria, glowed to the same extent under UV light suggesting that this method of detection may not be useful due to the high degree of background staining. b) Acrylamide based hydrogels show less background staining with DAPI than commercially available contact lenses. Non modified, non-functionalised commercial contact lenses and plain non-modified acrylamide gel were incubated with or without DAPI for 5 minutes, washed PBS 3 times and viewed under a UV light box. The commercial contact lens stained with DAPI, suggesting high background, whereas, when incubated with DAPI, the acrylamide gel did not have much background staining suggesting that using a different substrate for the polymer carrier might be advantageous in giving more options for dye selection. c) MTT staining can detect *S. aureus* after 1 hr incubation and *P.aeruginosa* and *C.albicans* after overnight incubation. Commercial contact lenses functionalised with vancomycin, polymxin and amphotericin polymers were incubated with or without 10^8^*S. aureus, P.aeruginosa* or *C.albicans* for one hour respectively. Lenses were washed 3 times with PBS and 0.5 mg ml^−1^ MTT solution added to the lenses for 1hr for *S. aureus*, overnight for *P.aeruginosa* and *C.albicans*.

Use of commercial contact lens materials also complicates the dye staining process used to the reveal bacterial or fungal isolates. The formulation used in the tested lenses responded to a range of dyes used for testing as shown in Fig. 6.

**Scheme 1:**
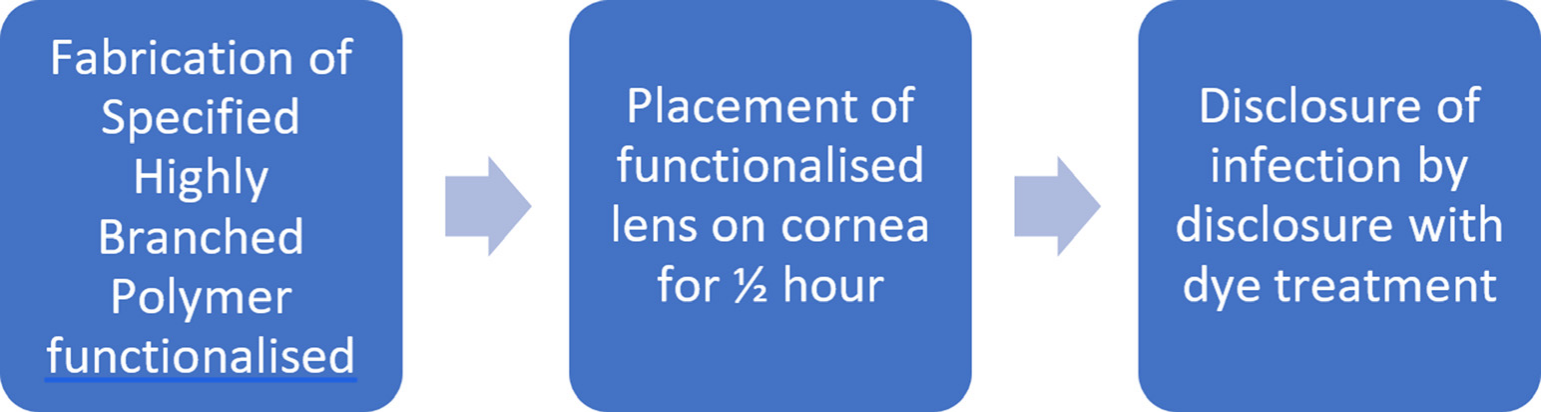
Rapid detection of corneal infections were disclosed within 30 minutes using pre-fabricated contact lens hydrogel swabs.

## Accuracy and sensitivity

The work shown in this paper shows a detection technique which requires both accuracy (ability to correctly disclose positively and to avoid false positives) and as low a limit sensitivity as possible. In all developmental laboratory work and pre-clinical in vivo animal trails carried out on infected rabbit corneas a 100% success rate was detected within a 30 minute exposure, with no toxigenic or immogenic response on the animals seen in the four weeks following testing [13] With laboratory testing the contact lenses indicated sensitivity to approximately 10^4^ CFU by luminescence on a plate reader, or even a discrimination between 10^1^ and 10^2^ CFU when analysed using a microscope. This data uses the optimized GMMA hydrogel backbone and, as this work has shown, will vary if other hydrogel bases or fluorescent dye systems are employed.

## Conclusions

The method describes here two methods for rapidly quantifying bacteria or fungal burden on surfaces using a hydrogel contact lens style swab and disclosing dyes. The first method has been shown to be highly selective and can be employed by laboratories with equipment to produce their own hydrogel sheets. The second shows a method of modifying any acid-functional hydrogel material (such as a commercial contact lens) to create a similar product with the knowledge that some specificity may be lost and additional work may be required to adjust the disclosing dyes following swabbing. However both approaches have been shown to function well at binding and then reporting bacterial burden on ex-vivo corneal surfaces.

The main advantage of this method compared to microbiological culture is rapid infection type identification within half an hour (gram positive, gram negative or fungal mode) which would provide a clinical indicator for tertiary care workers before providing a prescription. The materials can be fabricated and stored for several months meaning that following their distribution they can be employed in any remote setting as long as the required dyes (fluorescent vanc, FITC and calcofluor white) are provided as a disclosure kit.

## Declaration of Competing Interests

We gratefully acknowledge support for this research by the 10.13039/100010269Wellcome Trust which provided funding for Swift, Pinnock and Shivshetty (Grant 0998800/B/12/Z).
